# Eph/ephrin Function Contributes to the Patterning of Spinocerebellar Mossy Fibers Into Parasagittal Zones

**DOI:** 10.3389/fnsys.2020.00007

**Published:** 2020-02-13

**Authors:** Elizabeth P. Lackey, Roy V. Sillitoe

**Affiliations:** ^1^Department of Pathology & Immunology, Baylor College of Medicine, Houston, TX, United States; ^2^Department of Neuroscience, Baylor College of Medicine, Houston, TX, United States; ^3^Jan and Dan Duncan Neurological Research Institute at Texas Children’s Hospital, Houston, TX, United States; ^4^Development, Disease Models & Therapeutics Graduate Program, Baylor College of Medicine, Houston, TX, United States; ^5^Developmental Biology Graduate Program, Baylor College of Medicine, Houston, TX, United States

**Keywords:** cerebellum, mossy fiber, Purkinje cells, development, patterning, zebrinll

## Abstract

Purkinje cell microcircuits perform diverse functions using widespread inputs from the brain and spinal cord. The formation of these functional circuits depends on developmental programs and molecular pathways that organize mossy fiber afferents from different sources into a complex and precisely patterned map within the granular layer of the cerebellum. During development, Purkinje cell zonal patterns are thought to guide mossy fiber terminals into zones. However, the molecular mechanisms that mediate this process remain unclear. Here, we used knockout mice to test whether Eph/ephrin signaling controls Purkinje cell-mossy fiber interactions during cerebellar circuit formation. Loss of *ephrin-A2* and *ephrin-A5* disrupted the patterning of spinocerebellar terminals into discrete zones. Zone territories in the granular layer that normally have limited spinocerebellar input contained ectopic terminals in *ephrin-A2*^−/−^;*ephrin-A5*^−/−^ double knockout mice. However, the overall morphology of the cerebellum, lobule position, and Purkinje cell zonal patterns developed normally in the *ephrin-A2*^−/−^;*ephrin-A5*^−/−^ mutant mice. This work suggests that communication between Purkinje cell zones and mossy fibers during postnatal development allows contact-dependent molecular cues to sharpen the innervation of sensory afferents into functional zones.

## Introduction

Brain function requires precise input and output connections between neurons. In the cerebellar cortex, mossy fibers carry input from more than three dozen brain and spinal cord nuclei and terminate in a reproducible pattern of parasagittal zones within the granular layer (for reviews, see Sillitoe and Joyner, 2007; Apps and Hawkes, 2009; Voogd, 2014; Apps et al., 2018). The mossy fiber termination patterns of the spinocerebellar projections have been particularly well-characterized in adult and developing rodents (Grant, 1962; Voogd et al., 1969; Matsushita and Ikeda, 1980; Matsushita and Okado, 1981; Matsushita and Hosoya, 1982; Robertson et al., 1983; Arsénio Nunes et al., 1985; Gravel and Hawkes, 1990; Grishkat and Eisenman, 1995; Reeber et al., 2011b; Sillitoe, 2016; Luo et al., 2018). These studies showed that immature spinocerebellar mossy fibers segregate into the adult pattern of zones during postnatal development. However, the molecular mechanisms that guide the patterning of mossy fiber inputs into zones are not fully understood.

Data from different models led Sotelo and colleagues to propose that Purkinje cells act as organizer elements for the patterning of inputs to the cerebellar cortex (Wassef et al., 1985; Sotelo and Wassef, 1991). The Purkinje cell zonal architecture is now also thought to control zone formation of all other cerebellar components (Apps and Hawkes, 2009; Miterko et al., 2018). Developing and adult Purkinje cells express a wide variety of molecules in distinct parasagittal zones that could potentially mediate zone formation in the different cerebellar cell types (White and Sillitoe, 2013; Hawkes, 2014), while Purkinje cell zone formation itself is thought to be intrinsically driven (Leclerc et al., 1988; Wassef et al., 1990; Seil et al., 1995; Apps and Hawkes, 2009). Though adult mossy fibers terminate on granule cells, the adjacent Purkinje cell zonal boundaries have a reproducible relationship with those of mossy fiber terminals in the granular layer (Gravel and Hawkes, 1990; Matsushita et al., 1991; Ji and Hawkes, 1994; Quy et al., 2011; Luo et al., 2018) such that different Purkinje cell zones align with and/or subdivide neighboring mossy fiber zones. However, during postnatal development in the mouse and kitten, developing mossy fiber terminals form transient contacts with Purkinje cells before displacing to innervate their postsynaptic granule cell targets that subsequently invade the granular layer (Mason and Gregory, 1984; Takeda and Maekawa, 1989; Kalinovsky et al., 2011), potentially providing an opportunity for Purkinje cells to communicate directly with mossy fibers during circuit development. More recent work showed that the transient spinocerebellar mossy fiber contacts on Purkinje cells are refined to Purkinje cell zones at around postnatal day (P) 4 in the mouse (Sillitoe, 2016), suggesting that cell-cell contacts with Purkinje cells could be essential for the formation of mossy fiber parasagittal zones. This idea is consistent with data from mutant mice in which mossy fiber zones were disrupted in the absence of Purkinje cells but developed normally in the absence of granule cells (Arsénio Nunes et al., 1988). These findings suggest that mossy fiber patterning does not require synaptogenesis with their target granule cells but does require the presence of Purkinje cells, *via* an unknown mechanism. Analyses of mutant mice whose Purkinje cells are present in the cerebellum but lack organization into zones also show abnormal spinocerebellar termination patterns (Vogel et al., 1996; Sillitoe et al., 2010; Reeber et al., 2013; White et al., 2014), suggesting that the mechanism by which Purkinje cells influence mossy fiber patterning likely relies on the positional framework of Purkinje cell zones. Hawkes and colleagues ablated the spinocerebellar tract in the neonatal rat and showed that cuneocerebellar mossy fibers, which terminate in zones that interdigitate with the spinocerebellar zones, still respected the zonal boundaries and did not invade the “open” spinocerebellar territories of the granular layer despite the lack of competition (Ji and Hawkes, 1995). These data support a mechanism whereby mossy fibers recognize molecular cues in a zone. Sotelo and colleagues also proposed that the biochemical heterogeneity of Purkinje cell zones could reflect positional molecular cues that match with molecular cues on the incoming climbing fibers and mossy fibers; the effector molecules were, at the time, unknown but were postulated to act as guides for parasagittal zonation (Wassef et al., 1985; Sotelo and Wassef, 1991; Sotelo, 2004; Sotelo and Chédotal, 2005; Apps and Hawkes, 2009), perhaps through chemoaffinity (Sperry, 1963).

One family of molecules that could satisfy this role is the ephrin receptor tyrosine kinases (Eph) and ephrin molecules. *Eph/ephrin* genes encode membrane-bound molecules that mediate attraction and repulsion between cells (for reviews, see Flanagan and Vanderhaeghen, 1998; Wilkinson, 2001; Kania and Klein, 2016). The roles of the ephrin-A and EphA subfamilies as effector molecules that pattern neural circuit topography through chemoaffinity has been well-described in different sensory (Cheng et al., 1995; Drescher et al., 1995; Huffman and Cramer, 2007) and motor systems (Kania and Jessell, 2003; Iwasato et al., 2007). Analyses of Eph/ephrin expression in the developing chick and mouse have shown that the Purkinje cell layer and the external granular layer express ephrin-A ligands in the anterior and posterior cerebellum (Rogers et al., 1999; Karam et al., 2000; Saywell et al., 2014). Interestingly, immature granule cells in the external granular layer inhibit the growing mossy fibers from invading beyond the Purkinje cell layer (Manzini et al., 2006). Importantly, Bothwell and colleagues showed that ephrin-A2 expression and ephrin-A5 expression are arranged in parasagittal zones of Purkinje cells in the developing chick cerebellum (Karam et al., 2000) and related family members are also expressed in zones in the mouse cerebellum (Karam et al., 2002). Ephrin-A2 and ephrin-A5 are ligands for EphA receptors (Gale et al., 1996), and expression of EphA receptors has been documented in the pre-cerebellar nuclei where mossy fiber and climbing fiber inputs to the cerebellar cortex originate (Lin and Cepko, 1998; Rogers et al., 1999; Karam et al., 2000, 2002; Blanco et al., 2002; Nishida et al., 2002; Hashimoto et al., 2012; Saywell et al., 2014). *In vitro* work in the developing chick showed that overexpression of ephrin-A2 in the cerebellum repels climbing fiber inputs that express EphA receptors (Nishida et al., 2002). However, Bothwell and colleagues examined knockout mice that lack the *EphA4* gene expressed in Purkinje cells and reported that the Purkinje cell zones developed normally (Karam et al., 2002). Therefore, it is still unclear whether Eph/ephrin signaling mediates the formation of Purkinje cell zones or whether they control the patterning of cerebellar afferents *in vivo*. We hypothesized that zonal expression of the ephrin-A subfamily in Purkinje cells refines cell-cell contacts with mossy fiber terminals into parasagittal zones. The idea that specific Eph/ephrin molecules or combinations of the different subtypes could influence cerebellar patterning is appealing because an ephrin combinatorial code organizes visual maps (Cang et al., 2005). The likely functional redundancy established by the many overlapping Eph/ephrin subtypes in the cerebellum may require multiple genes to be deleted in order to observe a dramatic effect. Here, we used a combination of anterograde spinocerebellar tract-tracing and double knockout *ephrin-A2*^−/−^;*ephrin-A5*^−/−^ mice (Feldheim et al., 2000) to test the role of Eph/ephrin signaling in patterning spinocerebellar mossy fiber terminal fields into cerebellar parasagittal zones.

## Materials and Methods

### Animals

We performed all experiments in accordance with a protocol approved by the Institutional Animal Care and Use Committee at Baylor College of Medicine and the National Institutes of Health guidelines. We purchased *ephrin-A2^−/−^;*ephrin-A5*^−/−^* double knockout mice (Feldheim et al., 2000) from The Jackson Laboratory (*Efna2^tm1Jgf^* *Efna5^tm1Ddmo^*/J, Bar Harbor, ME, USA; #005992). We used a combination of C57BL/6J control mice purchased from The Jackson Laboratory (#000664) and littermate controls generated from breeding the alleles. We used both types of controls due to breeding difficulties of the mutant line. During breeding, we considered the day a vaginal plug was visible as embryonic day (E) 0 and the day of birth as P0. All mice were analyzed for cerebellar zonal patterns between 1 and 2 months of age.

### Neural Tract Tracing Using Sterile Surgery

We performed anterograde tracing of spinocerebellar afferents with WGA-Alexa 555 as we have previously described (Reeber et al., 2011a,b; Levy et al., 2017). WGA-Alexa 555 tracers travel quickly after injection, and they are robust when visualized in axons and terminals. Moreover, the Alexa fluorescent tag allows them to be easily combined with immunohistochemistry so that axon and terminal labeling can be examined in reference to neighboring cells. Briefly, we administered a 1 mg/kg dose of sustained-release buprenorphine and a 5 mg/kg dose of meloxicam by subcutaneous injections as preoperative analgesics. We anesthetized mice with Avertin (2,2,2-Tribromoethanol, Sigma-Aldrich, St. Louis MO, USA; #T48402) or with 1–4% isoflurane and administered a mixture of lidocaine and bupivacaine by intradermal injection as a local anesthetic. We made an incision in the skin above the lower thoracic-upper lumbar spinal cord. We cut the soft tissue connecting the T10 and T11 vertebral segments to expose the T13 and L1 spinal cord segments using the curvature of the spine as a guide (Harrison et al., 2013). We used a Nanoject II (Drummond Scientific, Broomall, PA, USA; #3-000-204) secured with a stereotaxic frame (David Kopf Instruments, Tujunga, CA, USA; Model 940) to pressure inject 0.2–1 μl of 2% WGA-Alexa 555 (Thermo Fisher Scientific, Waltham, MA, USA; #W32464) diluted in 0.1 M phosphate-buffered saline (PBS, Sigma-Aldrich; Cat #P4417; pH 7.4) just right of the dorsal spinal vein and approximately 1 mm below the dorsal surface of the spinal cord. We supplemented the tracer solution with 0.5% Fast Green (Sigma-Aldrich; #F7252) to visualize tracer injection during surgery. We applied antibiotic ointment and closed the incision with wound clips (Fine Science Tools, Foster City, CA, USA; #12032-07) and VetBond (3M, Maplewood, MN, USA; #1469SB). We placed 31M diet gel and hydrogel on the floor of the cage and carefully monitored the mice during the post-operative recovery period. We administered a 5 mg/kg dose of meloxicam by subcutaneous injection as a postoperative analgesic every 24 h and provided additional analgesic as needed. After a survival period of 48 h to allow the anterograde transport of WGA-Alexa 555 from the spinal cord to the cerebellum, we anesthetized the mice with Avertin and performed cardiac perfusion with PBS followed by 4% paraformaldehyde (PFA, pH 7.4) diluted in PBS. We sectioned and mounted brain and spinal cord tissue to image the tracer signal at the site of injection and in the terminals located in the cerebellum (see below).

### Tissue Preparation

We obtained free-floating frozen cut tissue sections as previously described (Reeber et al., 2011a,b; Levy et al., 2017). Briefly, we post-fixed brains and spinal cords for 24–48 h in 4% PFA and then cryoprotected the tissue stepwise in 15% and 30% sucrose solutions (diluted in PBS) at 4°C. We embedded the tissue in Tissue-Tek Optimal Cutting Temperature Compound (Sakura Finetek, Torrance, CA, USA; #4583) and froze the tissue at −80°C. We cut 40 μm sections on a cryostat at −20°C and collected them in PBS. We performed free-floating immunohistochemistry (see below) or immediately mounted sections on electrostatically coated slides with Fluoro-gel (Electron Microscopy Sciences, Hatfield, PA, USA; #17985-10).

### Immunohistochemistry

We performed free-floating immunohistochemistry as we have previously described (Sillitoe et al., 2010; Reeber et al., 2011b; White et al., 2014). Briefly, we blocked free-floating tissue sections for 1–2 h in 10% normal donkey serum (Sigma-Aldrich; #D9663) and 0.2% Triton-X 100 (Thermo Fisher Scientific #BP151-100) in PBS while gently shaking at room temperature, and then we incubated the free-floating tissue sections in primary antibodies for 16–18 h while shaking at room temperature. We used mouse monoclonal anti-zebrinII antibody (kind gift from Dr. Richard Hawkes, University of Calgary, Alberta, Canada) or rabbit polyclonal anti-Hsp25 antibody (Enzo Life Sciences, Farmingdale, NY, USA; #ADI-SPA-801-F) at concentrations of 1:500 diluted in blocking solution. After 3 × 5 min washes in PBS, we incubated the free-floating tissue sections in anti-mouse (Donkey anti-mouse IgG Alexa Fluor 488, Thermo Fisher Scientific #A21202) or anti-rabbit (Donkey anti-rabbit IgG Alexa Fluor 488, Thermo Fisher Scientific #A21206) Alexa fluorophore-conjugated secondary antibodies at concentrations of 1:1,500, again diluted in the blocking solution, for 2 h while shaking at room temperature. We then washed the sections 3 × 5 min in PBS before mounting on the electrostatically coated slides with Fluoro-gel.

### Microscopy

We captured photomicrographs of tissue sections with AxioCam MRm and MRc5 cameras (Zeiss, Oberkochen, DE) mounted on a Zeiss Axio Imager.M2 microscope. We acquired the images of tissue sections using Zeiss Zen software (2012 edition). We captured photomicrographs of whole-mount brains and spinal cords with Zeiss AxioCam MRm and MRc5 cameras mounted on a Zeiss Axio Zoom.V16 microscope. We acquired the whole-mount images using Zeiss AxioVision software (release 4.8). We pseudocolored WGA-Alexa 555 to magenta for better visualization. We imported the raw data into Adobe Photoshop CC and corrected the images for brightness and contrast levels.

### Image Analysis

**Figure 1:**
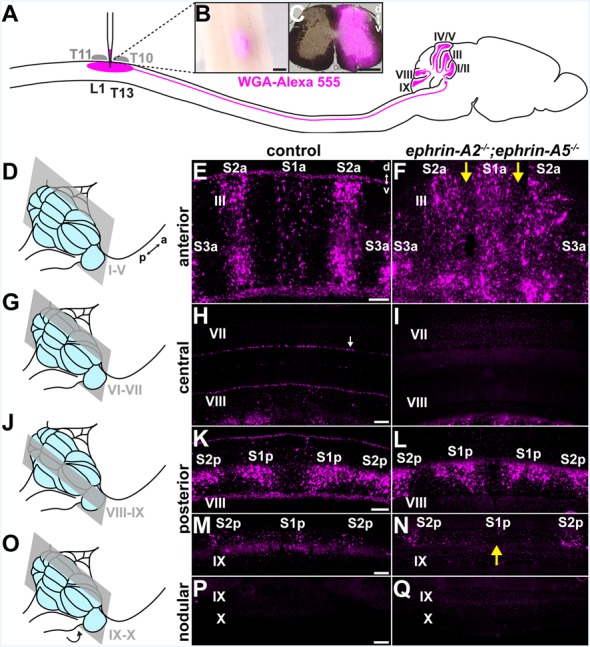
Spinocerebellar mossy fiber patterning is disrupted in *ephrin-A2*^−/−^;*ephrin-A5*^−/−^ mice. **(A)** Strategy for labeling spinocerebellar mossy fiber terminals in the cerebellar cortex of *ephrin-A2*^−/−^; *ephrin-A5*^−/−^ double knockout mice and control mice. We injected wheat germ agglutinin conjugated to Alexa fluorophores (WGA-Alexa 555) into the lower thoracic-upper lumbar spinal cord. Based on the curvature of the spine, we used vertebral segments T10 and T11 as landmarks to inject tracer into the underlying spinal cord segments of T13 to L1 (Harrison et al., 2013). **(B)** Whole-mount image of a dorsal view of a lower thoracic-upper lumbar spinal cord. A fluorescent image is overlaid on a bright-field image. The WGA-Alexa 555 tracer injection site is visible just lateral to the midline. a, anterior; p, posterior. Scale = 500 μm. **(C)** The A coronal section adjacent to a large injection site located in the lower thoracic-upper lumbar spinal cord. A fluorescent image is overlaid on a bright-field image. d, dorsal; v, ventral. Scale = 500 μm. **(D)** Schematic depicting a mouse brain with the cerebellum highlighted in blue and a coronal section through lobules I-V highlighted in gray. **(E)** Image of the WGA-Alexa 555 signal in the anterior cerebellum of a control mouse (*N* = 6). A midline parasagittal zone (S1a) and two zones lateral to the midline (S2a and S3a) are visible in lobule III. d, dorsal; v, ventral. Scale = 100 μm. **(F)** Image of the WGA-Alexa 555 signal in spinocerebellar mossy fibers in the anterior cerebellum of an *ephrin-A2*^−/−^;*ephrin-A5*^−/−^ double knockout mouse (*N* = 6). The boundaries of S1a and S2a in lobule III are less defined, and territories of the granular layer that have a limited termination of spinocerebellar mossy fiber terminals in control mice contain ectopic spinocerebellar mossy fiber terminals (yellow arrows). **(G)** Schematic depicting a mouse brain with the cerebellum highlighted in blue and a coronal section through lobules VI-VII highlighted in gray. **(H)** Image after WGA-Alexa 555 tracing to test for spinocerebellar mossy fibers in the central cerebellum of a control mouse (*N* = 6). Spinocerebellar mossy fiber terminals are not present in lobule VII. The background staining in the Purkinje cells is likely due to leakage of the WGA-Alexa 555 tracer from the cerebrospinal fluid that accumulates from the injection in the spinal cord (Sillitoe et al., 2010; white arrow). Scale = 100 μm. **(I)** Image after tracing to test for WGA-Alexa 555 signal in spinocerebellar mossy fibers in the central cerebellum of an *ephrin-A2*^−/−^;*ephrin-A5*^−/−^ double knockout mouse (*N* = 6). Spinocerebellar mossy fibers are not present in lobule VII. **(J)** Schematic depicting a mouse brain with the cerebellum highlighted in blue and a coronal section through lobules VIII and IX highlighted in gray. **(K)** Image of the WGA-Alexa 555 signal in spinocerebellar mossy fibers in the posterior cerebellum of a control mouse (*N* = 6). Two symmetrical pairs of parasagittal zones are visible in lobule VIII (S1p and S2p). Scale = 100 μm. **(L)** Image of the WGA-Alexa 555 signal in spinocerebellar mossy fibers in the posterior cerebellum of an *ephrin-A2*^−/−^;*ephrin-A5*^−/−^ double knockout mouse (*N* = 6). The two symmetrical pairs of parasagittal zones in lobule VIII are visible and relatively well-defined (S1p and S2p). **(M)** Image of the WGA-Alexa 555 signal in spinocerebellar mossy fibers in the posterior cerebellum of a control mouse (*N* = 6). A midline parasagittal zone and a zone lateral to the midline are visible in anterior lobule IX (S1p and S2p). Scale = 100 μm. **(N)** Image of the WGA-Alexa 555 signal in spinocerebellar mossy fibers in the posterior cerebellum of an *ephrin-A2*^−/−^;*ephrin-A5*^−/−^ double knockout mouse (*N* = 6). The midline parasagittal zone (S1p) and the zone lateral to the midline (S2p) are visible in anterior lobule IX. However, the S1p zone terminals are poorly organized compared to those in control mice (yellow arrow). **(O)** Schematic depicting a mouse brain with the cerebellum highlighted in blue and a coronal section through the nodular cerebellum (lobules posterior IX–X) highlighted in gray. The arrow indicates that lobule X is located underneath the posterior cerebellum, out of view. **(P)** Image after WGA-Alexa 555 tracing to examine for spinocerebellar mossy fibers in the nodular cerebellum of a control mouse (*N* = 6). Spinocerebellar mossy fibers are not present in posterior lobule IX or lobule X. Scale = 200 μm. **(Q)** Image after WGA-Alexa 555 tracing to examine for spinocerebellar mossy fibers in the nodular cerebellum of an *ephrin-A2*^−/−^;*ephrin-A5*^−/−^ double knockout mouse (*N* = 6). Spinocerebellar mossy fibers are not present in posterior lobule IX or lobule X.

We examined the WGA-Alexa 555 signal in matched coronal sections from the anterior, central, posterior, and nodular transverse domains (see Figure 1) based on the known regionalization and transitions between spinocerebellar mossy fiber zones (Grant, 1962; Voogd et al., 1969; Robertson et al., 1983; Gravel and Hawkes, 1990; Reeber et al., 2011b). To characterize the zones based on the previously described spinocerebellar termination pattern in rodents, we designated the spinocerebellar zones as “S” starting from the midline moving laterally as “S1, S2, + ….” We used an “a” or “p” to distinguish between the anterior zones (S1a, S2a, + ….) and the posterior zones (S1p, S2p, + ….) since the exact relationship between the spinocerebellar mossy fiber terminal fields in anterior and posterior zones remains unclear, though it has been demonstrated that individual spinal projection neurons can collateralize to terminate in the anterior and posterior cerebellar lobules (Heckroth and Eisenman, 1988; Luo et al., 2018). Therefore, it is unclear to what extent a given anterior mossy fiber zone is anatomically equivalent or linked to a posterior zone beyond sharing the originating fiber. Please refer to the Results for additional information about nomenclature. We observed occasional background WGA-Alexa 555 labeling in the molecular and Purkinje cell layers that we have previously observed with this technique due to leakage of WGA-Alexa 555 into the cerebrospinal fluid during surgery (Sillitoe et al., 2010). We further examined adjacent matched sections with immunohistochemistry to detect the Purkinje cells.

We measured the WGA-Alexa 555 signal intensity in the granular layer using the Plot Profile function in ImageJ, and we wrote custom MATLAB (MathWorks, Natick, MA, USA) code to calculate the intensity relative to the mean in the negative zones (lacking spinocerebellar terminals) for each genotype. The region of interest included S1a, S2a, and the negative zones between S1a and S2a in left and right lobule III, ±350 microns from the cerebellar midline. For all measurements, we calculated the intensity relative to the section mean to control for differences in intensity between sections. In order to calculate the relative intensities in the negative zones, we defined the boundaries between the zones as the positions where the second derivative of the intensity vector changed sign. Each boundary reflected a local minimum or maximum in the slope of the relative intensity vector along the mediolateral axis. We smoothed the data with a moving average filter when calculating the derivatives. We then plotted the boundary position values on the original unsmoothed relative intensity data and found that our approach reliably isolated the zones. We calculated the mean relative intensity in the negative zones for each genotype using the original unsmoothed data. We analyzed three sections per animal for a total of six negative zones per animal (*n*) and three animals per genotype (*N*). We performed all statistical analyses on the unsmoothed relative intensity data.

In order to measure the molecular layer thickness, we used the line measurement tool in ImageJ to measure the distance from the top of the Purkinje cell soma to the top of the Purkinje cell dendritic tree on images of coronal sections immunostained with the anti-zebrinII antibody. We obtained the measurements from the midline of posterior lobule VIII and anterior lobule IX in three sections per lobule for a total of six measurements per animal (*n*) and three animals per genotype (*N*).

For all statistical tests, we calculated the mean for each genotype and compared the genotypes using a two-tailed unpaired *t*-test with *N* as the number of animals and a significance threshold of *p* < 0.05. We reported the error as the standard error of the mean.

## Results

### Spinocerebellar Mossy Fiber Zones Are Disrupted in ephrin-A2^−/−^;*ephrin-A5*^−/−^ Mutant Mice

**Figure 2:**
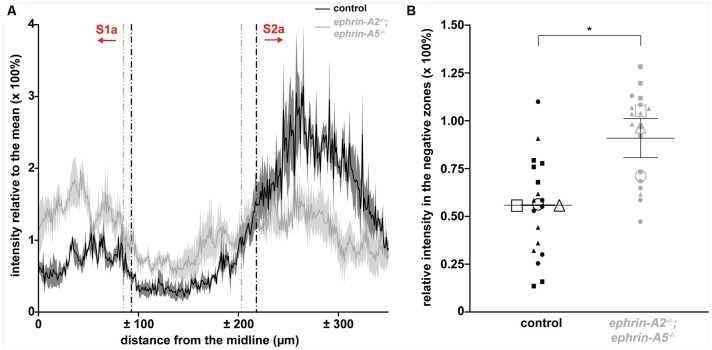
*ephrin-A2*^−/−^;*ephrin-A5*^−/−^ mice have ectopic spinocerebellar terminals. **(A)** Plot of the WGA-Alexa 555 relative intensity in the granular layer of lobule III for control and *ephrin-A2*^−/−^;*ephrin-A5*^−/−^ mice [*n* = 3 sections (six left/right negative zones) per animal, *N* = 3 animals per genotype]. The y-axis values indicate a ratio of the intensity to the mean intensity for the section, which directly represents a percentage of the mean intensity value. The x-axis values indicate the distance from the cerebellar midline as measured per pixel and scaled for μm per pixel. Vertical dotted lines indicate the boundaries of the negative zones between S1a and S2a calculated as where the second derivative of the smoothed intensity vector changes sign. The calculated zone boundaries are plotted on the original unsmoothed intensity data. Error bands indicate the standard error of the mean. **(B)** The WGA-Alexa 555 relative intensity in the negative zones between S1a and S2a is increased in the *ephrin-A2*^−/−^;*ephrin-A5*^−/−^ mice [control = 56.1% ± 0.21%; *ephrin-A2*^−/−^;*ephrin-A5*^−/−^ = 91.2% ± 10.3%; *n* = 6 negative zones per animal (small data points), *N* = 3 animals per genotype (large data points, each shape represents a different animal), *p* = 0.0267]. Error bars indicate the standard error of the mean. *p < 0.05.

We asked whether ephrin-A2/ephrin-A5 are effector molecules by which Purkinje cells organize mossy fiber zones downstream of the Purkinje cell zonal framework. If ephrin-A2 and ephrin-A5 are necessary for guiding mossy fibers into zones, then the loss of *ephrin-A2* and *ephrin-A5* should disrupt mossy fiber termination patterns. To test this, we used mice that lack the genes encoding ephrin-A2 and ephrin-A5 to examine the patterning of spinocerebellar terminal fields. The *ephrin-A2*^−/−^;*ephrin-A5*^−/−^ double knockout mice showed generally normal motor function with no gross abnormalities in locomotion, coordination, or balance (*N* = 6). We injected wheat germ agglutinin tracer conjugated to Alexa 555 fluorophores (WGA-Alexa 555) into the lower thoracic-upper lumbar spinal cord of *ephrin-A2*^−/−^;*ephrin-A5*^−/−^ double knockout mice (*N* = 6) and control mice (*N* = 6) to anterogradely trace and label spinocerebellar projections (Reeber et al., 2011a,b; Levy et al., 2017; Figure 1A) and then examined the labeled profiles in matched sections from the cerebellar cortex. We examined the WGA-Alexa 555 signal at the injection sites and found that we likely targeted spinocerebellar neurons arising from the dorsal nucleus of Clarke as well as neurons in laminae IV–VII and border cells of the upper thoracic to the lower lumbar spinal cord segments (Figures 1B,C). In the granular layer, we examined for the traced and labeled spinocerebellar mossy fibers in coronal sections (Figures 1D–Q). In order to characterize the traced and labeled spinocerebellar zones with reference to the previously characterized termination pattern (Grant, 1962; Voogd et al., 1969; Robertson et al., 1983; Gravel and Hawkes, 1990; Reeber et al., 2011b) for the purpose of this study, we designated the spinocerebellar zones as “S” starting from the midline moving laterally as “S1, S2, + ….” We used an “a” or “p” to distinguish between the anterior zones (S1a, S2a, + …) and the posterior zones (S1p, S2p, + …) because the relationship between the spinocerebellar mossy fiber terminals in anterior and posterior zones has not been fully defined, though single spinal neurons do collateralize to terminate in both anterior and posterior cerebellar lobules (Heckroth and Eisenman, 1988; Luo et al., 2018). In both mutant and control mice, we found that the traced spinocerebellar mossy fibers terminated in the granular layer of the anterior lobules (I–V; Figures 1D–F) and the posterior lobules (VIII-anterior IX; Figures 1J–N) and did not terminate in the central lobules (VI–VII; Figures 1G–I) or the nodular lobules (posterior IX–X; Figures 1O–Q). While *ephrin-A2*/*ephrin-A5* deletion did not disrupt the targeting of mossy fibers to the granular layer or to the correct lobules, it did disrupt the refinement of the parasagittal spinocerebellar zones. In the vermis of the anterior lobules, the traced spinocerebellar mossy fibers terminated in a midline zone (S1a) and two zones that are lateral to the midline (S2a and S3a; Figure 1E). We found that the deletion of *ephrin-A2* and *ephrin-A5* disrupted the mediolateral segregation of spinocerebellar terminal fields in the anterior zones (Figure 1F). Territories of the granular layer in lobule III that have limited spinocerebellar mossy fiber input in control mice contained ectopic spinocerebellar mossy fibers in the *ephrin-A2*^−/−^;*ephrin-A5*^−/−^ mutant mice (Figure 1F). Spinocerebellar mossy fiber terminals normally occupy complementary zones to the cuneocerebellar mossy fibers (Quy et al., 2011; Gebre et al., 2012). We observed labeled spinocerebellar terminals arranged into crude zones in *ephrin-A2*^−/−^;*ephrin-A5*^−/−^ mice, and their boundaries were less sharply defined due to the ectopic terminals that we observed to invade the adjacent granule cell territory that normally would be occupied by the cuneocerebellar terminals (Figure 1F). We detected the effect on mossy fiber zones in vermal lobule III but not in the zones of other anterior lobules or in the hemispheres where the known termination pattern is less clearly segregated in control mice (Supplementary Figure S1). We did not observe ectopic spinocerebellar mossy fibers terminating in the central lobules (VI–VII; Figures 1G–I). In the vermis of the posterior lobules, the overall pattern of the traced spinocerebellar mossy fiber zones in the double mutants was normal compared to that in controls, and the boundaries that define each zone were easily distinguished (Figures 1J–N). However, we observed poorly defined zonal clusters within the S1p of lobule IX (Figure 1N). We did not observe ectopic spinocerebellar mossy fibers terminating in the nodular lobules (posterior IX–X; Figures 1O–Q). In order to quantify the effect of *ephrin-A2*/*ephrin-A5* deletion on spinocerebellar mossy fiber zones, we measured the WGA-Alexa 555 signal intensity in the negative zones (lacking spinocerebellar terminals) of the granular layer between the S1a zone and the left and right S2a zones in lobule III (Figure 2A). We calculated the intensity relative to the mean to control for differences in intensity between sections, and we defined the boundaries between zones as the distances from the midline where the second derivative of the smoothed intensity vector changed the sign at each zone transition (Figure 2A). We found that the WGA-Alexa 555 relative intensity was increased in the negative zones of the granular layer between S1a and S2a in the mutants compared to that of the controls (control = 56.1% ± 0.21%; *ephrin-A2*^−/−^;*ephrin-A5*^−/−^ = 91.2% ± 10.3%; *n* = 6 negative zones per animal, *N* = 3 animals per genotype, *p* = 0.0267; Figure 2B). These results show that the genetic deletion of *ephrin-A2*/*ephrin-A5* caused a significantly increased number of spinocerebellar mossy fibers to terminate in the zones between S1a and S2a that normally lack spinocerebellar terminals in controls. These data suggest that Eph/ephrin signaling is required for refining spinocerebellar mossy fiber zones. Our data also show that *ephrin-A2* and *ephrin-A5* are not required for specifically targeting spinocerebellar axons and terminals into the granular layer or to the correct lobules in the anterior-posterior axis of the cerebellum.

### ephrin-A2 and ephrin-A5 Are Not Required for the Formation of Purkinje Cell Zones

**Figure 3:**
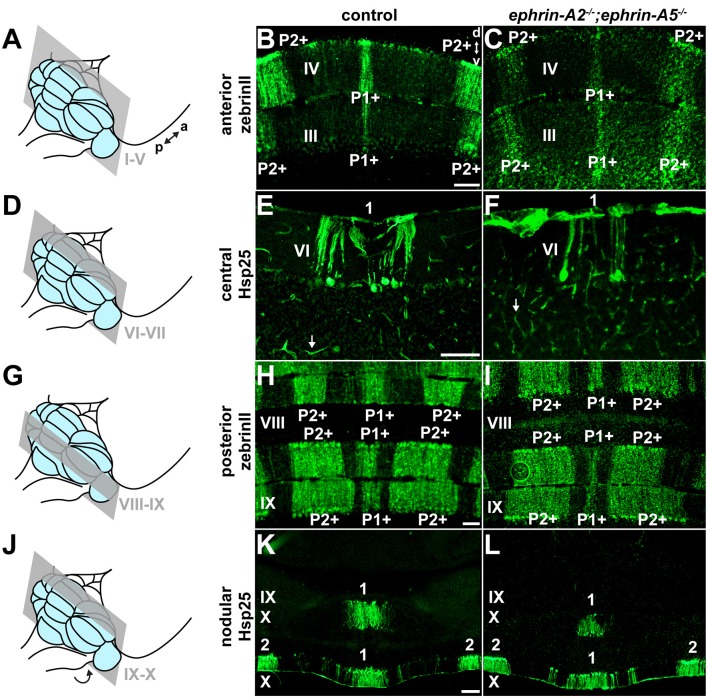
*ephrin-A2* and *ephrin-A5* are not required for the formation of Purkinje cell zones. **(A)** Schematic depicting a mouse brain with the cerebellum highlighted in blue and a coronal section through the anterior cerebellum (lobules I-V) highlighted in gray. a, anterior; p, posterior. **(B)** Image of zebrinII expression in the anterior cerebellum of a control mouse (*N* = 6). A medial zone of Purkinje cells (P1+) and a zone lateral to the midline (P2+) are visible. d, dorsal; v, ventral. Scale = 100 μm. **(C)** Image of zebrinII expression in the anterior cerebellum of an *ephrin-A2*^−/−^;*ephrin-A5*^−/−^ double knockout mouse (*N* = 6). The medial zone of Purkinje cells (P1+) and the zone lateral to the midline (P2+) are visible and correctly organized. **(D)** Schematic depicting a mouse brain with the cerebellum highlighted in blue and a coronal section through the central cerebellum (lobules VI-VII) highlighted in gray. **(E)** Image of Hsp25 expression in the central cerebellum of a control mouse (*N* = 6). The focus is on the medial zone of Purkinje cells (1). Blood vessels are also immunoreactive for Hsp25 (white arrow). Scale = 100 μm. **(F)** Image of Hsp25 expression in the central cerebellum of an *ephrin-A2*^−/−^;*ephrin-A5*^−/−^ double knockout mouse (*N* = 6). The medial zone of Purkinje cells (1) has sharp boundaries. Blood vessels are also immunoreactive for Hsp25 (white arrow). **(G)** Schematic depicting a mouse brain with the cerebellum highlighted in blue and a coronal section through the posterior cerebellum (lobules VIII and anterior IX) highlighted in gray. **(H)** Image of zebrinII expression in the posterior cerebellum of a control mouse (*N* = 6). A medial zone of Purkinje cells (P1+) and a zone lateral to the midline (P2+) are visible. Scale = 100 μm. **(I)** Image of zebrinII expression in the posterior cerebellum of an *ephrin-A2*^−/−^;*ephrin-A5*^−/−^ double knockout mouse (*N* = 6). The medial zone of Purkinje cells (P1+) and the zone lateral to the midline (P2+) are correctly organized. **(J)** Schematic depicting a mouse brain with the cerebellum highlighted in blue and a coronal section through the nodular cerebellum (lobules posterior IX–X) highlighted in gray. The arrow indicates that lobule X is located underneath the posterior cerebellum and is therefore out of view. **(K)** Image of Hsp25 expression in the nodular cerebellum of a control mouse (*N* = 6). A medial zone of Purkinje cells (1) and a zone lateral to the midline (2) are labeled. Scale = 200 μm. **(L)** Image of Hsp25 expression in the nodular cerebellum of an *ephrin-A2*^−/−^;*ephrin-A5*^−/−^ double knockout mouse (*N* = 6). The medial zone of Purkinje cells (1) and the zone lateral to the midline (2) have a normal distribution pattern.

We next asked whether ephrin-A2/ephrin-A5 are also required for the formation of Purkinje cell zones. If ephrin-A2/ephrin-A5 mediate the formation of Purkinje cell zones, then we would expect the loss of *ephrin-A2* and *ephrin-A5* to disrupt Purkinje cell zonal patterning. To test whether loss of *ephrin-A2* and *ephrin-A5* disrupts Purkinje cell zones, we used immunohistochemistry to detect molecular markers of Purkinje cell zones in order to label and examine their expression patterns in matched coronal sections from each of the four transverse domains of the cerebellar cortex (anterior, central, posterior, and nodular; Ozol et al., 1999; Sillitoe et al., 2005) in *ephrin-A2*^−/−^;*ephrin-A5*^−/−^ mice (*N* = 6) and control mice (*N* = 6; Figures 3A–L). In the anterior (I–V) and posterior (VIII-anterior IX) lobules of control mice, zebrinII/AldolaseC marks a striking array of Purkinje cell zones (Brochu et al., 1990; Gravel and Hawkes, 1990; Ji and Hawkes, 1994; Ozol et al., 1999). In contrast, the small 25 kDa heat shock protein (Hsp25) marks Purkinje cell zones in the central (VI–VII) and nodular (posterior IX–X) lobules of control mice, where zebrinII is uniformly expressed in all Purkinje cells (Armstrong et al., 2000). Anti-Hsp25 immunohistochemistry also weakly labels blood vessels and ependymal cells throughout the cerebellum (Armstrong et al., 2000). We tested zebrinII and Hsp25 because their Purkinje cell expression patterns are a sensitive readout of developmental and disease-associated defects that disrupt Purkinje cell zones (Sillitoe et al., 2008; White et al., 2016). Along the mediolateral axis of vermal lobules I–V (Figure 3A), zebrinII is expressed in a medial zone of Purkinje cells (P1+) and a zone that is lateral to the midline (P2+; Figure 3B). We found that the deletion of *ephrin-A2* and *ephrin-A5* did not disrupt the pattern of anterior Purkinje cell zones (Figure 3C). We also observed occasional weak labeling of Purkinje cell axon collaterals in the granular layer with zebrinII immunohistochemistry in mutants and controls, as previously reported (Brochu et al., 1990). Posteriorly in the central lobules VI and VII (Figure 3D), Hsp25 is expressed by a medial zone of Purkinje cells (1; Figure 3E) and two zones that are lateral to the midline (the complete Hsp25 map is described in Armstrong et al., 2000). We found that the deletion of *ephrin-A2* and *ephrin-A5* does not disrupt the central Purkinje cell zones (Figure 3F). Moving more posteriorly into the vermis of lobules VIII and anterior IX (Figure 3G), zebrinII expression is again patterned, with a medial zone (P1+) flanked by two zones (P2+ and P3+; Figure 3H). We found that deletion of *ephrin-A2* and *ephrin-A5* does not disrupt the posterior Purkinje cell zones (Figure 3I), Note that for clarity we have only described the most medial and prominent zebrinII zones in the anterior and posterior lobules (for a full description see Ozol et al., 1999; Sillitoe and Hawkes, 2002). In the nodular lobules (Figure 3J), Hsp25 is expressed by a medial zone of Purkinje cells (1) and a zone lateral to the midline (2) in posterior lobule IX and similarly in a medial zone (1) and a zone lateral to the midline (2) in lobule X (Figure 3K; Armstrong et al., 2000). We found that deletion of *ephrin-A2* and *ephrin-A5* does not disrupt the nodular Purkinje cell zones (Figure 3L). For two well-described Purkinje cell markers, zebrinII and Hsp25, we showed that deletion of *ephrin-A2* and *ephrin-A5* did not disrupt the Purkinje cell zones in the anterior, central, posterior, or nodular lobules. In the mutants, the zonal expression patterns were restricted to the expected Purkinje cell subsets and arranged in the same distribution with clear zone boundaries as observed in control mice. The data suggest that *ephrin-A2* and *ephrin-A5* are not required for the formation or maintenance of sharp Purkinje cell zones in mice.

### The Relationship Between Purkinje Cell Zones and Spinocerebellar Mossy Fiber Zones Is Disrupted in ephrin-A2^−/−^; *ephrin-A5*^−/−^ Mutant Mice

**Figure 4:**
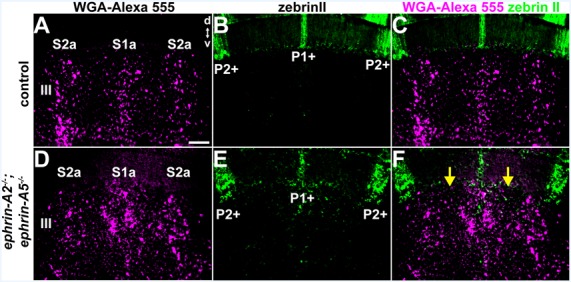
The relationship between Purkinje cell zones and spinocerebellar mossy fiber zones is disrupted in *ephrin-A2*^−/−^;*ephrin-A5*^−/−^ mutant mice. **(A–C)** Image of the WGA-Alexa 555 signal and zebrinII expression in the anterior cerebellum of a control mouse (*N* = 6). The patterning of the spinocerebellar mossy fiber zones has a systematic relationship to the map defined by the Purkinje cell zones. d, dorsal, v, ventral. Scale = 100 μm. **(D–F)** Image of the WGA-Alexa 555 signal and zebrinII expression in the anterior cerebellum of an *ephrin-A2*^−/−^;*ephrin-A5*^−/−^ double knockout mouse (*N* = 3). The relationship between spinocerebellar terminal field zones (S1a and S2a) and the boundaries of the Purkinje cell zones (P1+ and P2+) is disrupted due to the poorly defined spinocerebellar zone boundaries (yellow arrows).

We examined the relationship between the pattern of WGA-Alexa 555 anterogradely labeled spinocerebellar mossy fiber terminal fields and the pattern of Purkinje cell zones that were marked using zebrinII immunohistochemistry in *ephrin-A2*^−/−^;*ephrin-A5*^−/−^ mice (*N* = 3) and control mice (*N* = 6). In control mice, the boundaries of the mossy fiber terminal fields have a reproducible relationship with Purkinje cell zones, which match or subdivide the afferent zones (Gravel and Hawkes, 1990; Matsushita et al., 1991; Ji and Hawkes, 1994; Reeber et al., 2011b; Figures 4A–C). We found that while zebrinII expression in the cerebellar cortex of *ephrin-A2*^−/−^;*ephrin-A5*^−/−^ double knockout mice is restricted to Purkinje cells similar to the distribution of zones in control mice, the relationship between spinocerebellar terminal fields and how far they extend beyond the boundaries of Purkinje cell zones is disrupted (Figures 4D–F). The granular layer contained ectopic spinocerebellar terminals adjacent to zebrinII-negative Purkinje cell zones that normally align with mossy fibers originating from the external cuneate nucleus (Gebre et al., 2012; Figures 4D–F). These data suggest that the topographical relationship between Purkinje cell zones and the mossy fiber subsets that normally reside below them requires Eph/ephrin signaling.

### Cerebellar Gross Morphology and Cerebellar Cortical Thickness Are Unaltered in Adult ephrin-A2^−/−^/*ephrin-A5*^−/−^ Mutant Mice

**Figure 5:**
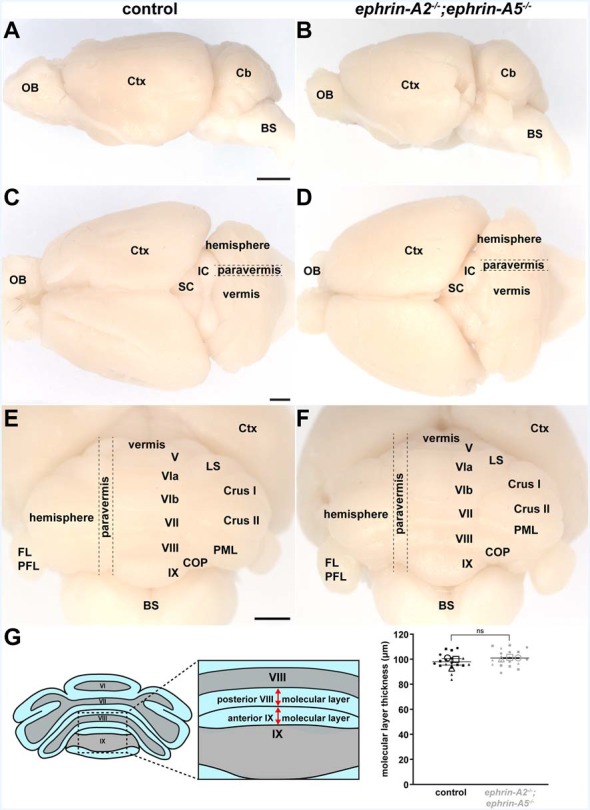
Cerebellar lobule position and cerebellar cortical thickness are unaltered in adult *ephrin-A2^−/−^/*ephrin-A5*^−/−^* mutant mice. **(A)** Whole-mount image of a lateral view of a control mouse brain (*N* = 6). OB, olfactory bulb; Ctx, cerebral cortex; Cb, cerebellum; BS, brain stem. Scale = 2 mm. **(B)** Whole-mount image of a lateral view of an *ephrin-A2*^−/−^;*ephrin-A5*^−/−^ double knockout mouse brain (*N* = 6). The cerebellum is in the correct location and proportional to the rest of the brain. **(C)** Whole-mount image of a dorsal view of a control mouse brain (*N* = 6). The gross mediolateral subdivisions of the cerebellum are visible (vermis, paravermis, hemisphere). OB, olfactory bulb; Ctx, cerebral cortex; SC, superior colliculus; IC, inferior colliculus. Scale = 1 mm. **(D)** Whole-mount image of a dorsal view of an *ephrin-A2*^−/−^;*ephrin-A5*^−/−^ double knockout mouse brain (*N* = 6). The gross mediolateral subdivisions of the cerebellum are fully represented and in their correct locations (vermis, paravermis, hemisphere). **(E)** Whole-mount image of a posterior view of a control mouse brain (*N* = 6). The cerebellar lobules are visible. Note that lobules I–IV and X of the vermis are located underneath the other lobules and out of view in this orientation. Ctx, cerebral cortex; LS, lobulus simplex; PML, paramedian lobule; COP, copula pyramidis; FL/PFL, flocculus and paraflocculus; BS, brain stem. Scale = 1 mm. **(F)** Whole-mount image of a posterior view of an *ephrin-A2^−/−^;*ephrin-A5*^−/−^* double knockout mouse brain (*N* = 6). The main lobules are fully represented and in their correct locations. **(G)** Quantification of the molecular layer thickness measured from lobules VIII and IX. Molecular layer thickness is unaltered in the mutant mice [control = 97.9 μm ± 2.55 μm; *ephrin-A2*^−/−^;*ephrin-A5*^−/−^ = 100.9 μm ± 0.373 μm; *n* = 6 measurements per animal (small data points), *N* = 3 animals per genotype (large data points, each shape represents a different animal), *p* = 0.3088]. Error bars indicate the standard error of the mean. ns = not significant.

One possible explanation for the defective mapping of spinocerebellar terminals in the *ephrin-A2*^−/−^;*ephrin-A5*^−/−^ double knockout mice is that the cerebellar cortical layers may have developed abnormally, which could mean that spinocerebellar mossy fibers found their correct positions but in different locations. Purkinje cells express *ephrin-A2* and *ephrin-A5* in the positions of future lobules along the anteroposterior axis of the embryonic cerebellum before the lobules form (Rogers et al., 1999; Karam et al., 2000), further raising the question of whether they have a role in shaping the cerebellum. We, therefore, tested whether *ephrin-A2/ephrin-A5* are required for the formation of cerebellar morphological features that could interfere with the proper establishment of afferent termination patterns. We examined the gross morphology of *ephrin-A2*^−/−^;*ephrin-A5*^−/−^ mouse brains compared to control brains (*N* = 6 for each genotype). We found that the shape of the cerebellum, based on the surface structural identity of the 10 lobules (Larsell, 1970), was normal in *ephrin-A2*^−/−^/*ephrin-A5*^−/−^ mutant mice (Figures 5A–F). The cerebellum and the 10 lobules were fully represented and in their correct locations (Figures 5A–F). To examine whether there were more subtle morphological changes to the laminar structure of the cerebellar cortex, we measured the thickness of the molecular layer. Molecular layer thickness is a sensitive and straightforward measure for developmental and disease-associated defects that disrupt Purkinje cell dendrites or the placement of Purkinje cells into a monolayer (Hansen et al., 2013; White et al., 2014, 2016; White and Sillitoe, 2017). Such defects could be predicted to influence the patterning of mossy fibers, likely through their direct contacts (Mason and Gregory, 1984; Sillitoe, 2016). We did not detect a difference in molecular layer thickness between *ephrin-A2*^−/−^;*ephrin-A5*^−/−^ mice and control mice (control mean = 97.9 μm ± 2.55 μm; *ephrin-A2*^−/−^;*ephrin-A5*^−/−^ mean = 100.9 μm ± 0.373 μm; *n* = 6 measurements per animal, *N* = 3 animals per genotype, *p* = 0.3088; Figure 5G). These data suggest that *ephrin-A2* and *ephrin-A5* are not required for establishing the basic cerebellar foliation plan, cerebellar cortical lamination, or for the general expansion of Purkinje cell dendrites.

## Discussion

Multiple studies have provided compelling evidence that Purkinje cells may act as organizer elements for the patterning of incoming cerebellar afferents (Wassef et al., 1985; Sotelo and Wassef, 1991; Ji and Hawkes, 1995; Sotelo, 2004; Sotelo and Chédotal, 2005; Sillitoe and Joyner, 2007; Apps and Hawkes, 2009; White and Sillitoe, 2013). Additional data have pointed to Eph/ephrin signaling as a potential molecular mechanism by which Purkinje cells could guide afferent terminals into zones (Cheng et al., 1995; Drescher et al., 1995; Lin and Cepko, 1998; Rogers et al., 1999; Karam et al., 2000, 2002; Blanco et al., 2002; Nishida et al., 2002; Saywell et al., 2014). In order to investigate the role of Eph/ephrin signaling in the formation of mossy fiber terminal zones, we performed anterograde neural tract-tracing of spinocerebellar mossy fibers in *ephrin-A2*^−/−^;*ephrin-A5*^−/−^ double knockout mice and examined the topography of labeled terminal fields in the cerebellar cortex. We found that loss of *ephrin-A2* and *ephrin-A5* disrupted the parasagittal patterning of spinocerebellar mossy fibers. Loss of *ephrin-A2* and *ephrin-A5* did not disrupt the organization of Purkinje cell zones or the basic morphology of the cerebellum. These data suggest that Eph/ephrin signaling is required for the patterning of spinocerebellar mossy fiber zones and, more broadly, that an abnormal Purkinje cell zonal map *per se* is not required for insults in Eph/ephrin signaling to consequently disrupt the precision of mossy fiber patterning.

Our data address a distinction between the molecular mechanisms that form parasagittal zones in Purkinje cells vs. mossy fibers. Purkinje cell patterning has been thought to control the topography of all other cerebellar components, with the idea that they accomplish this *via* intercellular communication mediated by cell-to-cell contact and/or molecular cues. We show that loss of specific Eph/ephrin molecular signals leaves the Purkinje cell map intact but nevertheless alters spinocerebellar afferent patterns. How then do Purkinje cells control afferent mapping? We speculate that Purkinje cell zones are not disrupted by deleting the *ephrin-A2* and *ephrin-A5* genes or by deleting the *EphA4* gene (Karam et al., 2002) because there are multiple stages of constructing the complete zonal module, which would include patterning of afferents and efferents around the Purkinje cell. We suggest that the genes encoding patterned Eph/ephrin positional cues in Purkinje cells are expressed as factors for executing the Purkinje cell program that shapes the module around an existing plan of zones—at those early stages, the module is likely exclusively made up of Purkinje cells. In this scenario, patterned Eph/ephrin combinations would function as effector molecules for matching incoming circuit projections with Purkinje cell zones, but these specific Eph/ephrin codes would not be required for forming the Purkinje cell zones themselves. This argument is supported by the timing of Purkinje cell zonal development. Viral-mediated marking of Purkinje cells at the time of their birth, between E10-E13 (Miale and Sidman, 1961), demonstrates an early assembly of zones (Hashimoto and Mikoshiba, 2003) which are further defined by gene expression starting at ~E14 (Oberdick et al., 1993; Millen et al., 1995; Larouche et al., 2006; Yaguchi et al., 2009; Wilson et al., 2011; Fujita et al., 2012; Vibulyaseck et al., 2017). There is strong evidence that the establishment of Purkinje cell zones is dependent on the *early B-cell factor 2* (*Ebf2*) gene that encodes a non-basic helix-loop-helix transcription factor and the homeobox-containing *Engrailed* (*En1/2*) genes. *Ebf2* plays a role in establishing the zebrinII-positive vs. negative identity of Purkinje cells (Croci et al., 2006; Chung et al., 2008), and *En1/2* are required for the early stages of patterning Purkinje cell zones (Baader et al., 1999; Sillitoe et al., 2008). Interestingly, *En1/2* acts upstream of *ephrin-A2* and *ephrin-A5* in the tectum to establish positional cues and guide the patterning of afferents from the retina that express *Eph* receptors (Logan et al., 1996; Shigetani et al., 1997). In the cerebellum, loss of *ephrin-A2* and *ephrin-A5* in Purkinje cells could lead to afferent termination defects because the absence of these cues affects the full composite of signals that define the cerebellar internal map, which would cause a failure in the ability of the mossy fibers to interpret the “zip code.” However, it is also possible that the many other Eph/ephrin molecules expressed in the cerebellum during development (Lin and Cepko, 1998; Rogers et al., 1999; Karam et al., 2000, 2002; Blanco et al., 2002; Nishida et al., 2002; Saywell et al., 2014) compensate for a potential role of ephrin-A2 and/or ephrin-A5 in establishing Purkinje zones. Indeed, future work would need to resolve the exact timing of the onset of Eph/ephrin expression in different embryonic cerebellar neurons in order to fully appreciate how these molecules influence patterning. In any case, altering the Purkinje cell map has proven very difficult with several molecular and injury methods, and likely reflects the intrinsic control of Purkinje cell patterning (Apps and Hawkes, 2009). It is, therefore, striking that loss of *ephrin-A2* and *ephrin-A5* disrupts spinocerebellar mossy fiber zones but not the Purkinje cell molecular code since the relationship between afferent identity and Purkinje cell molecular identity is resistant even to experimental manipulations that dramatically alter cerebellar morphology (Vogel and Prittle, 1994; Vig et al., 2005; Reeber et al., 2013). We showed that the relationship between Purkinje cell molecular zone identity and the mossy fiber termination pattern is disrupted in the absence of *ephrin-A2* and *ephrin-A5* and is not the result of a gross morphologic displacement of circuitry. These data lead us to ask whether the refinement of mossy fiber zones by ephrin-A2/ephrin-A5 is specific to the spinocerebellar subset or generalizes to other mossy fiber inputs. We suspect that a given set of Purkinje cell zones would guide multiple cerebellar inputs into zones depending on the molecular tags expressed at the axons/terminals. For instance, spinocerebellar and cuneocerebellar terminals likely share some molecular targeting signals, whereas the more posteriorly located vestibular mossy fiber afferent terminals could use at least some unique cues compared to the anteriorly projecting afferents. It is also possible that at a finer level, even the different cerebellar-projecting cell classes from the spinal cord (Clarke’s column, border cells, etc.) could use different Eph/ephrin molecules for patterning, which could explain the lobule-specific and overall circumscribed effects that we observed. Therefore, we speculate that dedicated, and probably combinatorial, Eph/ephrin signaling mechanism(s) could contribute to coordinating the organization of inputs and outputs of Purkinje cells during circuit formation.

Despite the reproducible defects we observed in spinocerebellar zonal targeting, a map, albeit altered, did form, and individual clusters of terminals were represented in the mutants. During nervous system development, the processes of axon guidance, target recognition, and map formation are controlled by a growing list of overlapping molecular mechanisms including Netrin/Unc/DCC, Slit/Robo, Semaphorins/Plexins, and the different Cadherin family members. Therefore, the partial segregation of spinocerebellar mossy fibers we observed in *ephrin-A2^−/−^;*ephrin-A5*^−/−^* mice could be due to the overlapping expression and function of other molecules in the cerebellum, such as cell-adhesion molecules (Arndt et al., 1998; Luo et al., 2004), additional Eph/ephrin family members (Lin and Cepko, 1998; Rogers et al., 1999; Karam et al., 2000, 2002; Blanco et al., 2002; Nishida et al., 2002; Saywell et al., 2014), or perhaps direct guidance by *En1/2* (Brunet et al., 2005). The loss of *ephrin-A2* or *ephrin-A5* could also cause more subtle or synaptic level changes to spinocerebellar mossy fiber topography that were not detected by the methods used here. We also expect that the loss of *ephrin-A2* or *ephrin-A5* could also disrupt the development of other components of the cerebellar circuit that were not tested here—for example, the different classes of interneurons. Because spinocerebellar mossy fiber zones have not been examined in *ephrin-A2* or *ephrin-A5* single knockout mice, our findings in the double knockout mice do not distinguish whether either gene is essential for refining mossy fiber zones independent of the other gene. However, there is a high level of redundancy in the Eph/ephrin family of signaling molecules (Gale et al., 1996; Feldheim et al., 2000), and we postulate that there is not a single master regulator of mossy fiber zone formation. Future experiments will hopefully reveal how ephrin-A2 and ephrin-A5, in concert with other molecules and pathways, precisely coordinate the attraction and repulsion of intrinsic and extrinsic fibers into precise cerebellar zonal maps.

## Conclusion

The cerebellum is organized around a pleated array of parasagittal zones. Purkinje cells are the central component of zones. Recent work has shown that Purkinje cell zones have distinct neuronal firing properties, which could determine how they control different motor and non-motor behaviors. However, the mechanisms that assemble zones are still unclear. There is a long-standing hypothesis that perhaps Purkinje cell zones provide the platform upon which all other cerebellar components establish their topography. The activity of a molecular cue that mediates cell-to-cell communication would satisfy this hypothesis. Here, we provide evidence that Eph/ephrin signaling contributes to spinocerebellar mossy fiber zones. We also show that while Purkinje cells may indeed guide incoming afferents and shape their terminal field patterns, they likely employ cell intrinsic cues to first set up their own zones and then express cues to direct the fibers. We anticipate that the full molecular profile for cues that generate mossy fibers zones includes a large number of proteins with diverse functions.

## Data Availability Statement

All datasets generated for this study are included in the article/Supplementary Material.

## Ethics Statement

The animal study was reviewed and approved by the Institutional Animal Care and Use Committee at Baylor College of Medicine.

## Author Contributions

EL and RS designed the experiments, performed the experiments, analyzed the data, wrote the article, and edited the article.

## Conflict of Interest

The authors declare that the research was conducted in the absence of any commercial or financial relationships that could be construed as a potential conflict of interest.

## Supplementary Material

FIGURE S1 WGA-Alexa 555 tracing of spinocerebellar mossy fibers. **(A)** Representative image of the WGA-Alexa 555 signal in lobules I/II of a control mouse (*N* = 6). d, dorsal, v, ventral. Scale = 200 μm. **(B)** Representative image of the WGA-Alexa 555 signal in lobules I/II of an *ephrin-A2*^−/−^;*ephrin-A5*^−/−^ mouse (*N* = 6). **(C)** Representative image of the WGA-Alexa 555 signal in lobules IV/V of a control mouse (*N* = 6). d, dorsal, v, ventral. Scale = 100 μm. **(D)** Representative image of the WGA-Alexa 555 signal in lobules IV/V of an *ephrin-A2*^−/−^;*ephrin-A5*^−/−^ mouse (*N* = 6). **(E)** Representative image of the WGA-Alexa 555 signal in the copula pyramidis of a control mouse (*N* = 6). Scale = 200 μm. **(F)** Representative image of the WGA-Alexa 555 signal in the copula pyramidis of an *ephrin-A2*^−/−^;*ephrin-A5*^−/−^ mouse (*N* = 6).
