# Supplementary figures and images for: Eph/ephrin Function Contributes to the Patterning of Spinocerebellar Mossy Fibers Into Parasagittal Zones
Source: Front Syst Neurosci. 2020 Feb 13;14:7. doi: 10.3389/fnsys.2020.00007 (PMC7033604; doi:10.3389/fnsys.2020.00007)

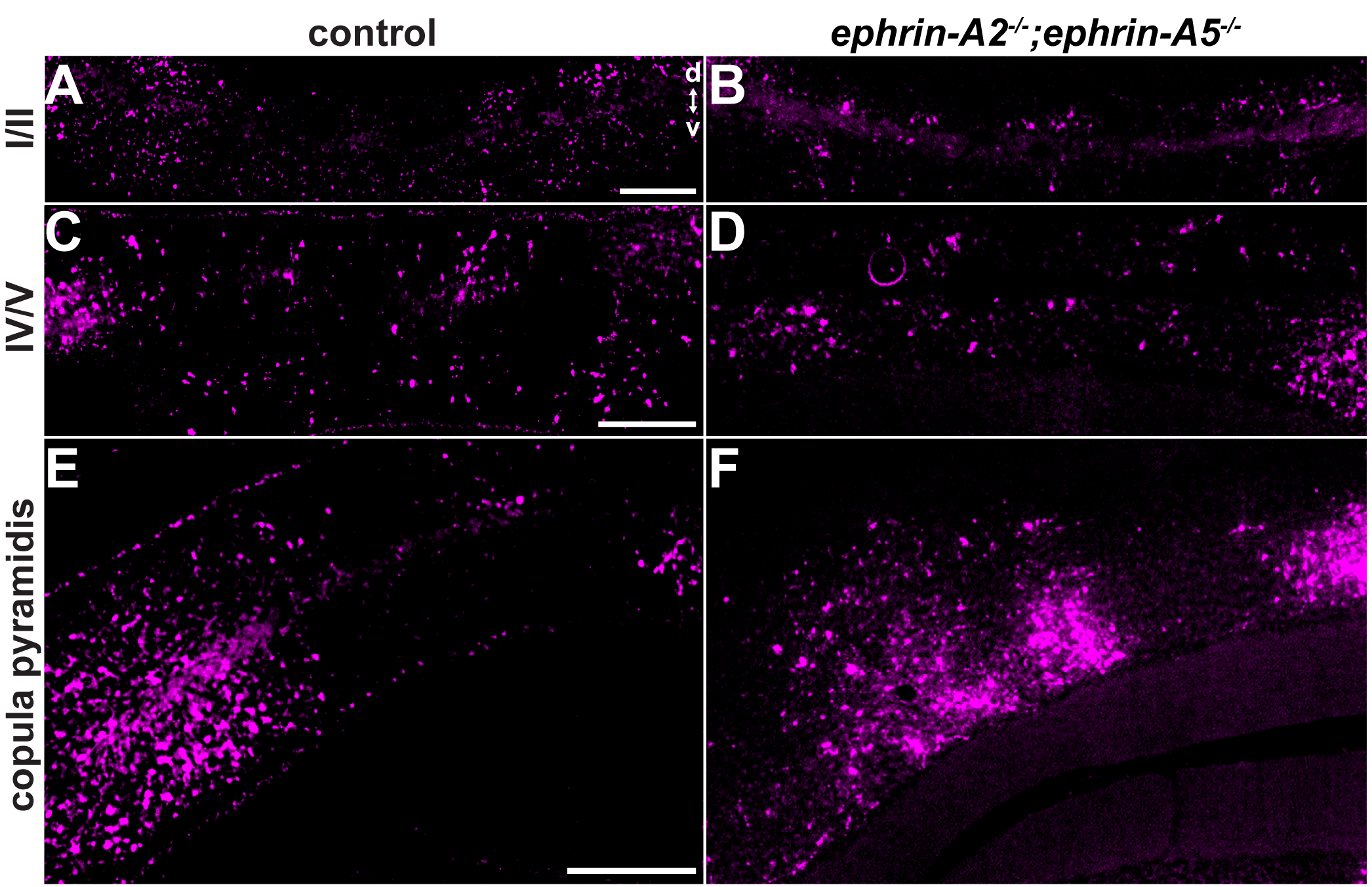

Supplement: FIGURE S1 — WGA-Alexa 555 tracing of spinocerebellar mossy fibers. (A) Representative image of the WGA-Alexa 555 signal in lobules I/II of a control mouse (N = 6). d, dorsal, v, ventral. Scale = 200 μm. (B) Representative image of the WGA-Alexa 555 signal in lobules I/II of an ephrin-A2−/−;ephrin-A5−/− mouse (N = 6). (C) Representative image of the WGA-Alexa 555 signal in lobules IV/V of a control mouse (N = 6). d, dorsal, v, ventral. Scale = 100 μm. (D) Representative image of the WGA-Alexa 555 signal in lobules IV/V of an ephrin-A2−/−;ephrin-A5−/− mouse (N = 6). (E) Representative image of the WGA-Alexa 555 signal in the copula pyramidis of a control mouse (N = 6). Scale = 200 μm. (F) Representative image of the WGA-Alexa 555 signal in the copula pyramidis of an ephrin-A2−/−;ephrin-A5−/− mouse (N = 6). [file Image_1.TIF]
